# Sequence-Based Discovery Demonstrates That Fixed Light Chain Human Transgenic Rats Produce a Diverse Repertoire of Antigen-Specific Antibodies

**DOI:** 10.3389/fimmu.2018.00889

**Published:** 2018-04-24

**Authors:** Katherine E. Harris, Shelley Force Aldred, Laura M. Davison, Heather Anne N. Ogana, Andrew Boudreau, Marianne Brüggemann, Michael Osborn, Biao Ma, Benjamin Buelow, Starlynn C. Clarke, Kevin H. Dang, Suhasini Iyer, Brett Jorgensen, Duy T. Pham, Payal P. Pratap, Udaya S. Rangaswamy, Ute Schellenberger, Wim C. van Schooten, Harshad S. Ugamraj, Omid Vafa, Roland Buelow, Nathan D. Trinklein

**Affiliations:** Teneobio, Inc., Menlo Park, CA, United States

**Keywords:** monoclonal human antibodies, transgenic rodent, rearranged light chain, somatic hypermutation, deep sequencing, antibody repertoire, humanized rodent

## Abstract

We created a novel transgenic rat that expresses human antibodies comprising a diverse repertoire of heavy chains with a single common rearranged kappa light chain (IgKV3-15-JK1). This fixed light chain animal, called OmniFlic, presents a unique system for human therapeutic antibody discovery and a model to study heavy chain repertoire diversity in the context of a constant light chain. The purpose of this study was to analyze heavy chain variable gene usage, clonotype diversity, and to describe the sequence characteristics of antigen-specific monoclonal antibodies (mAbs) isolated from immunized OmniFlic animals. Using next-generation sequencing antibody repertoire analysis, we measured heavy chain variable gene usage and the diversity of clonotypes present in the lymph node germinal centers of 75 OmniFlic rats immunized with 9 different protein antigens. Furthermore, we expressed 2,560 unique heavy chain sequences sampled from a diverse set of clonotypes as fixed light chain antibody proteins and measured their binding to antigen by ELISA. Finally, we measured patterns and overall levels of somatic hypermutation in the full B-cell repertoire and in the 2,560 mAbs tested for binding. The results demonstrate that OmniFlic animals produce an abundance of antigen-specific antibodies with heavy chain clonotype diversity that is similar to what has been described with unrestricted light chain use in mammals. In addition, we show that sequence-based discovery is a highly effective and efficient way to identify a large number of diverse monoclonal antibodies to a protein target of interest.

## Introduction

Human monoclonal antibodies (mAbs) are a promising and rapidly growing class of therapeutic molecules for various disease indications (1, 2). A significant technical achievement in creating therapeutic antibodies was the generation of transgenic mice (3, 4) and rats (5, 6) that express fully human Ig loci. Such systems enable the development of fully human mAbs with reduced risk of antidrug immune reactions in human patients. An additional class of transgenic rodents has also been created that express a full heavy chain repertoire with a fixed rearranged light chain (7, 8). Toward this end, we created a novel transgenic Sprague-Dawley rat strain called OmniFlic that expresses a fixed human kappa light chain in combination with a chimeric IgH locus including human V_H_s, Ds, J_H_s, and rat C_H_s in a triple Ig KO background (9). The human IgH V genes were covered by two bacterial artificial chromosomes (BACs): BAC6-VH3-11 containing the region spanning from VH4-39 to VH3-23 followed by VH3-11 and BAC3 containing the region spanning from VH3-11 to VH6-1 (811L16 RPCI-11) as described by Osborn et al. (9).

The use of OmniFlic has several advantages for developing therapeutic antibodies. Most significantly, the common light chain simplifies development of bispecific antibodies since a common light chain can be used with independently derived heavy chains that bind to different protein targets (10). Furthermore, the common light chain is a fully germline sequence with no predicted sequence liabilities thus decreasing downstream development risks.

Fixed light chain animal models also present a unique system for studying heavy chain repertoire diversity, somatic hypermutation (SHM), and antigen-specific binding in the context of a common light chain. Because VDJ/VJ recombination and SHM generate the diversity of the variable regions of the heavy and light chains independently in conventional antibodies it is difficult to determine whether the heavy chain or light chain or both are contributing to antigen binding. In fixed light chain animals, we can attribute antigen-binding specificity exclusively to the sequence of the heavy chain variable region. Thus our functional analysis of the heavy chain repertoire is not confounded by contributions of light chain variation.

In this study, we used next-generation sequencing to characterize the complete germinal center B-cell repertoires of 75 OmniFlic animals immunized with 9 different antigens. To evaluate binding specificity of fixed light chain mAbs (FlicAbs), we also expressed and measured antigen binding for 2,560 FlicAbs. Taken together, our analysis of heavy chain sequence repertoires demonstrates that VH gene usage in OmniFlic animals is similar to VH usage in natural human repertoires (11), and that OmniFlic animals produce a large number of diverse VDJ clonotypes found in germinal center B-cells at levels comparable to wild type mammals (12, 13). Furthermore, large-scale binding analysis of expressed FlicAbs proteins shows that antigen-specific binding activity follows patterns of SHM similar to what is seen in conventional antibodies (14).

## Results

### Summary of Design and Analysis

We immunized a total of 75 OmniFlic animals, each with 1 of 9 different human recombinant protein antigens. We harvested two draining lymph nodes from each animal and analyzed each lymph node separately for a total of 150 independent samples.

**Figure 1:**
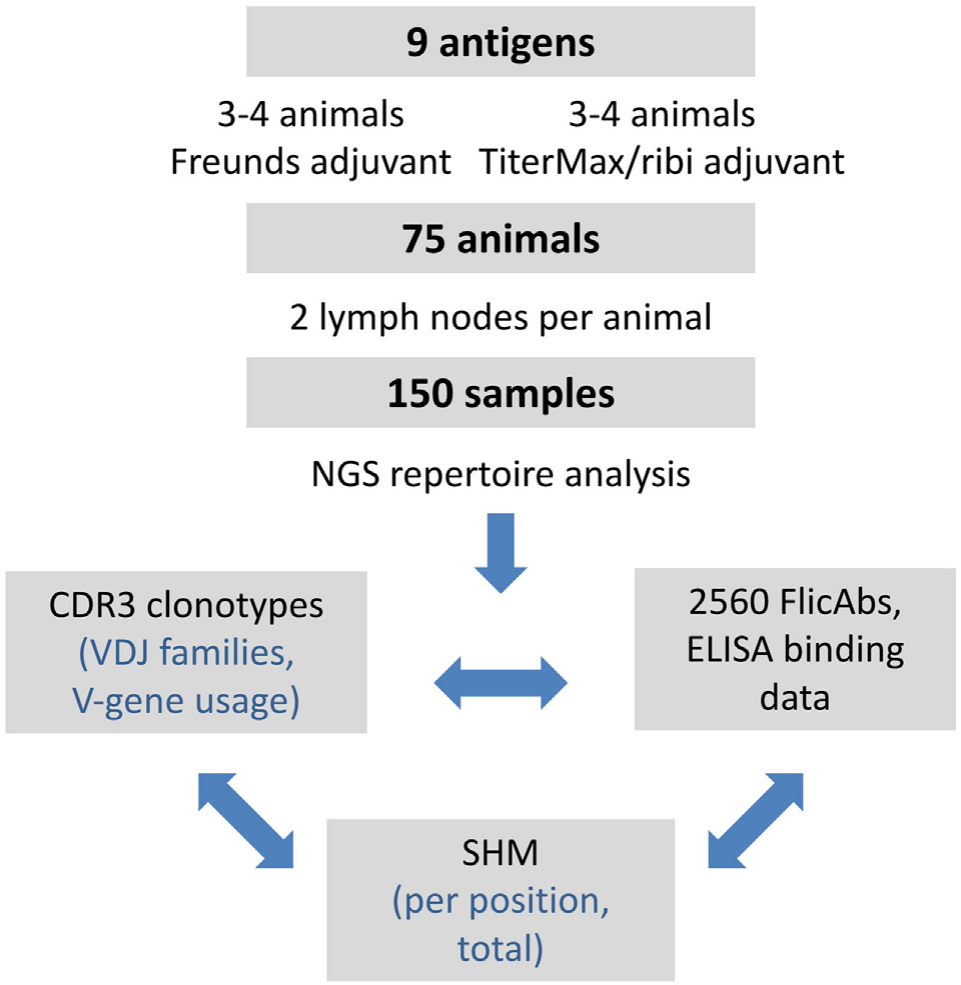
Summary of experimental design and analysis. The overall design of the project and subsequent analyses is shown. We performed a series of immunizations with 9 different antigens and included 75 different OmniFlic animals. We harvested 2 lymph nodes from each animal for a total of 150 different samples. Our analysis was focused on three areas: VDJ clonotype diversity by NGS, somatic hypermutation by NGS, and antigen binding by ELISA.

We focused our analysis of the repertoire data on quantifying IGHV gene usage, measuring VDJ clonotype diversity between lymph nodes and between animals, and measuring patterns of SHM. In additional to the repertoire analysis, we expressed a total of 2,560 monoclonal FlicAbs, measured protein binding by ELISA, and analyzed functional binding data in the context of clonotype size and SHM. The overall study design is summarized in Figure 1.

### Clonotype Sequence Diversity and IGHV-Gene Usage

The naïve B-cell repertoire of an organism is created by the random recombination of V, D, and J genes at the heavy chain locus and the recombination of V and J genes at the light chain locus (15). Naïve B-cells are exposed to antigen in germinal centers of lymphoid organs, and antigen-specific B-cells are positively selected for in the germinal centers (16). A primary goal of our project was to estimate the diversity of heavy chain VDJ clonotypes in germinal center B-cells undergoing affinity maturation due to antigen exposure. Since the heavy chain CDR3 of a B-cell is defined by the V–D–J recombination event in the naïve B-cell (17, 18), we used sequence similarity in the CDR3 region to define clonotype lineages. We accomplished this by clustering CDR3 sequences from the NGS repertoire data based on 80% sequence similarity to allow for variation introduced by SHM. Previous studies have shown that 80% sequence similarity of the CDR3 protein sequence is an effective threshold for identifying members of a clonotype (19). Then, we calculated the number of unique CDR3 clonotypes in each lymph node. Within each animal, we looked for CDR3 clonotypes that appear in both lymph nodes to estimate the frequency of B-cell migration between lymph nodes. Finally, we compared the total set of CDR3 clonotypes between all of the animals to measure the uniqueness of each animal’s repertoire.

**Figure 2:**
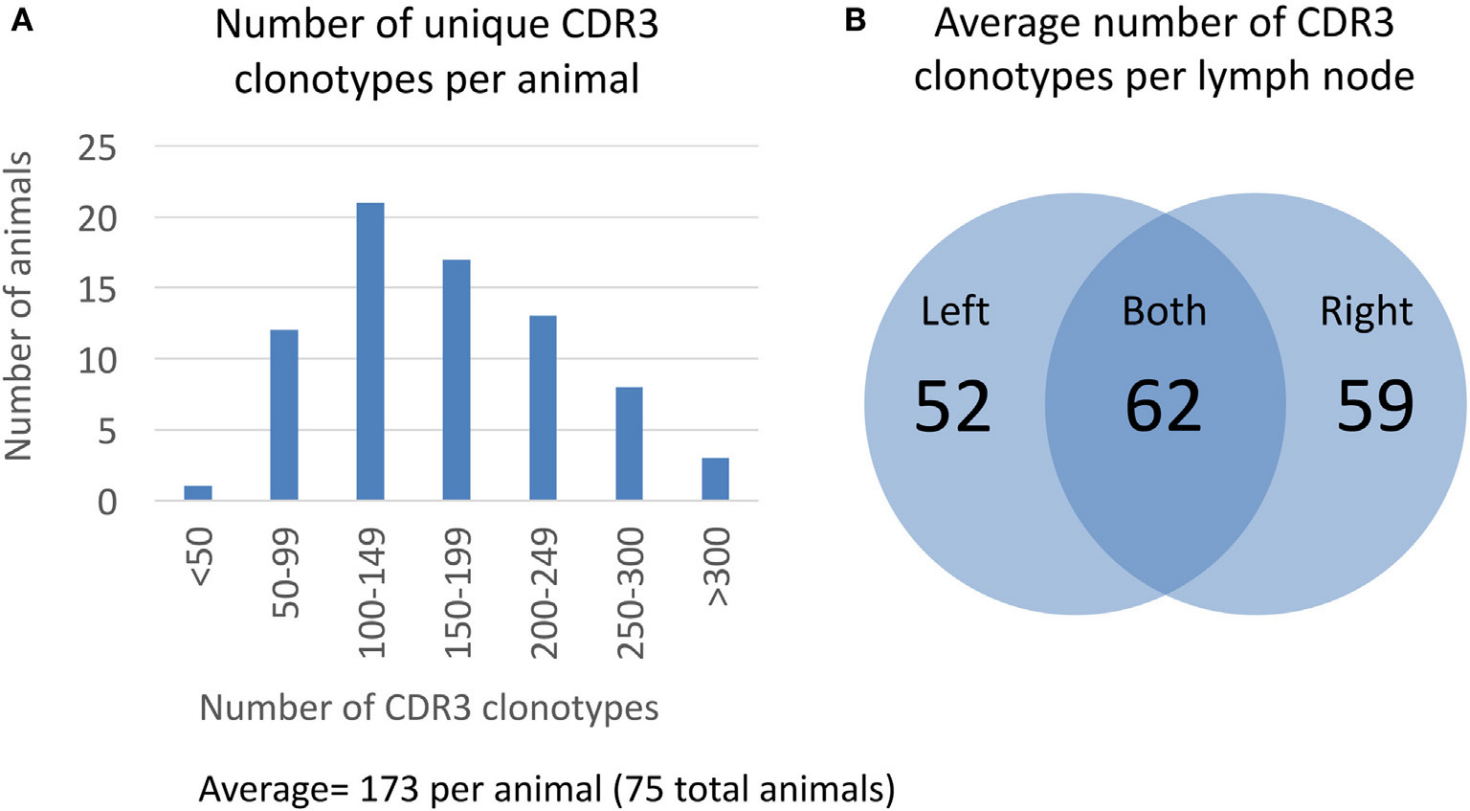
Clonotype diversity in 75 OmniFlic animals. **(A)** Distribution of CDR3 clonotypes per animal from a total of 75 OmniFlic animals. The number of unique CDR3 clonotypes per animal ranged from 47 to 433 with a mean of 173. **(B)** Overlap of CDR3 clonotypes between lymph nodes of the same animal. The 173 average CDR3 clonotypes per animal, 62 are common to both lymph nodes, and the remaining 111 are unique to 1 lymph node, on average.

After clustering CDR3 protein sequences based on 80% similarity, we measured the overall distribution of number of unique CDR3 clonotypes per animal (Figure 2). The average number of unique CDR3 clonotypes per animal is 173 with a minimum of 47 and a maximum of 433 (Figure 2A). Based on our analysis of technical sequencing replicates of the same lymph node sample, we estimate that our sequencing depth identifies 85% of the total CDR3 clonotypes from an animal, so the total average number of unique CDR3 clonotypes is estimated to be 204 (173/0.85, see Section “Materials and Methods” for details on sequencing depth analysis). This sequence-based measure of clonotype diversity is consistent with earlier work that estimated the size of the human polyclonal repertoire to be in the order of 100 B cell clones (20). Interestingly, approximately half of the CDR3 clonotypes from a given lymph node are not found in the contralateral lymph node from the same animal. On average, only 62 of 173 unique CDR3 clonotypes are found in both lymph nodes of an animal, whereas 52 and 59 CDR3 clonotypes are unique to the left and right lymph nodes, respectively (Figure 2B). This suggests that the majority of germinal center B-cells remain within a single lymph node.

To determine the overlap of CDR3 clonotypes between animals, we compared each animal’s total list of CDR3 clonotypes to that of every other animal resulting in an all-by-all analysis (Figure S1 in Supplementary Material). The average percent overlap of CDR3 clonotypes between two animals immunized with the same antigen is 17.2%. By contrast, the average percent overlap of CDR3 clonotypes between two animals immunized with different antigens is 2.2%. These data suggest that sequence convergence occurs between animals that are immunized with the same antigen. However, environmental factors may also play a role in apparent convergence due to the fact that animals receiving the same antigen were housed together and immunized at the same time.

In addition to measuring the total number of distinct CDR3 clonotypes, we also wanted to compare the total number of sequences within the different of CDR3 clonotypes within an animal (otherwise referred to as sequence polarization). Based on the initial affinity of the B-cell receptor when exposed to antigen, higher affinity CDR3 clonotypes are selected for and will show increased levels of cell proliferation and Ig expression, accumulating mutations as they divide (21). Thus, the CDR3 clonotypes represented by the largest number of sequences may be produced by a large number of high-expressing germinal center B-cells due to positive antigen selection. Figure S2 in Supplementary Material summarizes the fraction of total sequences contained in the top 10 CDR3 clonotypes based on sequence abundance per animal. The top ranked, most abundant CDR3 clonotype ranges from less than 10% of all sequences in some animals to greater than 80% of all sequences in other animals. This observation correlates with the total number of unique CDR3 clonotypes in that animal such that animals with fewer unique sequences tend to be the most polarized. Low clonotype diversity with strong polarization may result from strong selection for a small number of immunodominant epitopes on the target antigen (22).

**Figure 3:**
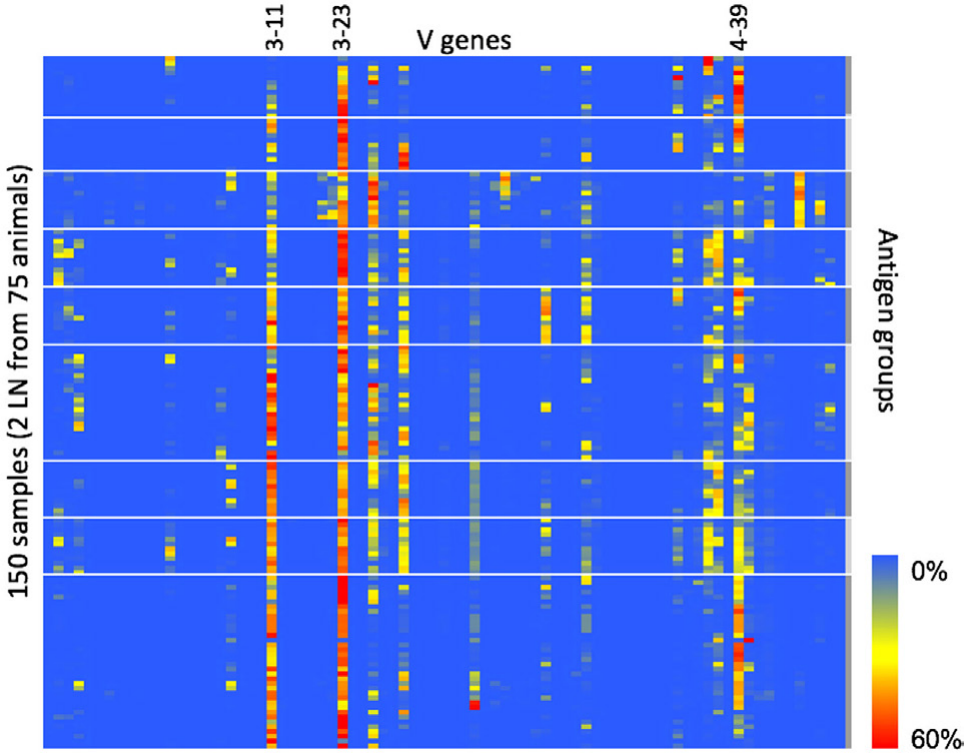
IGHV-gene usage in 150 different samples. IGHV-gene usage is summarized in this heat map. Heavy chain variable regions most commonly found in natural human repertoires are also the most frequently used IGHV genes in OmniFlic. Each column is one human heavy chain V gene defined by IMGT. Each row is one lymph node sample. A blue box indicates 0% frequency of that V gene in that sample. A red box indicates 60% frequency of that V gene in that sample. Heavy white lines denote boundaries between the nine antigen groups.

Finally, since CDR3 clonotype diversity is partially determined by V-gene usage, we wanted to measure the IGHV-gene usage within each animal to see if the fixed light chain restricted IGHV-gene usage compared with natural human B-cell repertoires. For each sequence in the heavy chain repertoire data, we assigned V-gene use based on the highest similarity to a germline reference sequence. The IGHV-gene usage is summarized in Figure 3. Of the 79 total human V genes, 24 were found at a frequency of 5% or greater in at least 1 animal. The most frequently used V genes (IGHV3-11, IGHV3-30, IGHV3-23, and IGHV4-39) correspond to the most frequently used V genes that were paired with IGKV3-15 in natural human repertoires (11). Therefore, despite the fixed light chain, OmniFlic uses a diverse set of human V genes, and V-gene usage corresponds to what is observed in natural human repertoires.

### Isolation of Monoclonal FlicAbs and Determination of Antigen-Specific Binding

**Figure 4:**
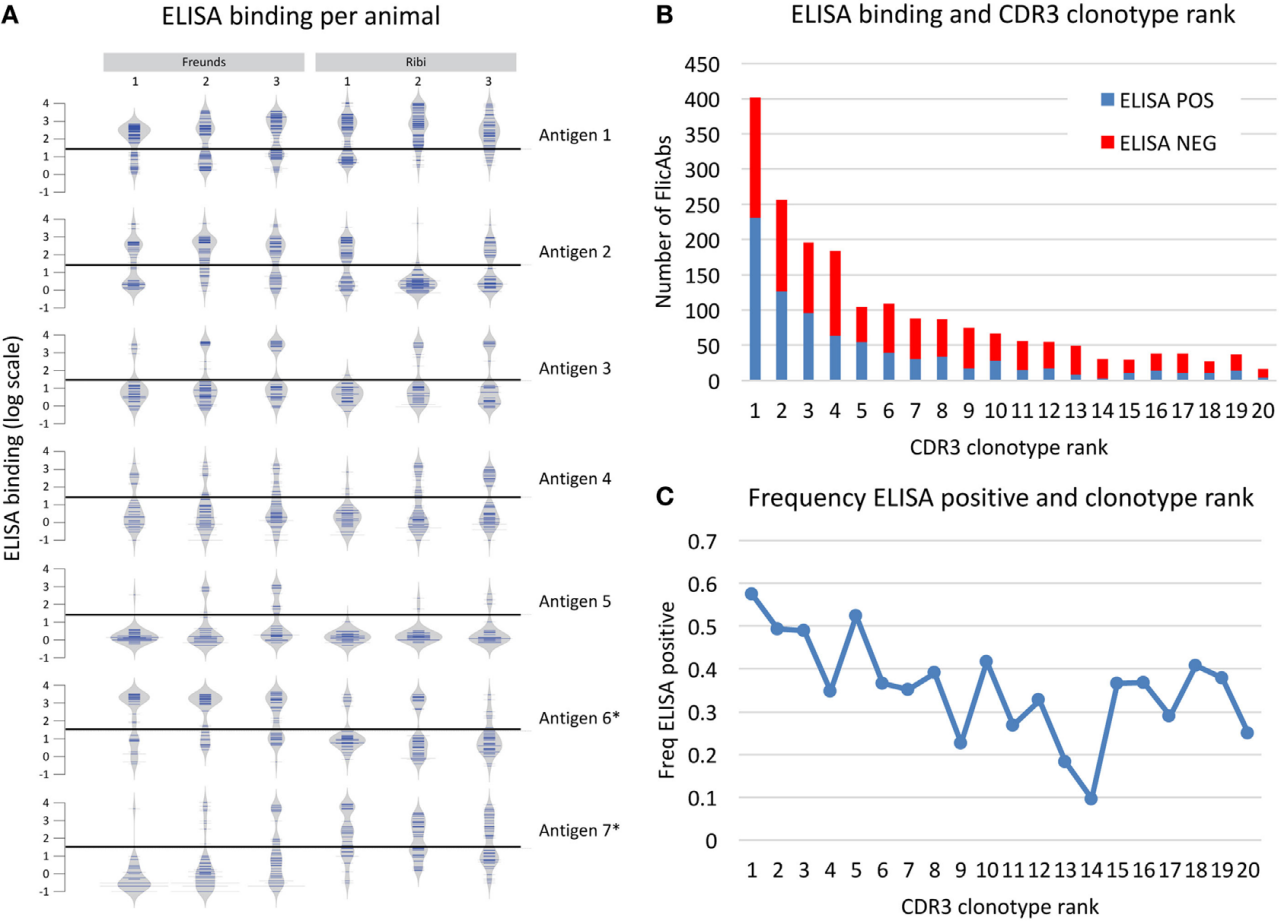
Antigen binding measured for 2,560 monoclonal FlicAbs. **(A)** Individual bean plots show the distribution of antigen-binding signal for 42 representative animals (6 animals immunized with 7 different antigens). The *y*-axis shows log transformed ELISA binding signal (fold-over background), and the horizontal line marks the threshold used for calling a FlicAb antigen positive or negative. The left three vertical columns are animals immunized with Freund’s adjuvant, and the right three columns are animals immunized with Ribi adjuvant. Each row is a different antigen. **(B)** The distribution of clonotype abundance (CDR3 clonotype rank) of the FlicAbs tested are shown as stacked bars where the red bar indicates ELISA negative FlicAbs and the blue indicates ELISA-positive FlicAbs. The *y*-axis indicates total FlicAb counts, and the *x*-axis category designates the abundance rank of the CDR3 clonotype. **(C)** The frequency of ELISA-positive FlicAbs categorized by abundance rank is shown. The *y*-axis indicates the frequency of ELISA positive, and the *x*-axis category designates the abundance rank of the CDR3 clonotype.

Based on the heavy chain sequence repertoire determined by NGS and the analysis of CDR3 clonotypes, we selected a total of 2,560 unique FlicAbs for expression and binding assessment. This set of FlicAbs represents an average of 34 unique heavy chain variable region sequences per animal. We chose representative sequences from a diverse set of CDR3 clonotypes from each animal. We also chose multiple sequences from individual clonotypes in proportion to the total size of the clonotypes in an attempt to sample evenly across the repertoire while covering as much clonotype diversity as possible. For each of the 2,560 VH sequences, we assembled a heavy chain expression vector and individually co-transfected each construct with a light chain expression vector containing the germline-configured fixed light chain sequence expressed by OmniFlic. After transient expression of the 2,560 individual FlicAb proteins in mammalian cells, we harvested FlicAb-containing cell culture supernatant and measured antigen-specific and off-target binding by ELISA. We used negative control supernatants in each ELISA experiment to normalize binding signal as fold-over background (Figure 4). After normalizing the ELISA binding results, the dataset for each antigen showed a bi-modal distribution with 10-fold-over background serving as a threshold that effectively separated ELISA positive from ELISA negative binding for each antigen (see Figure 4A).

Overall, 1,008 of the 2,560 FlicAbs (39%) were antigen specific. One of the goals in this study was to examine sequence features of the antigen-specific class of FlicAbs compared with the FlicAbs that did not bind antigen. The antigen-specific binding of these FlicAbs is driven exclusively by mutation of the heavy since all of these antibodies were expressed with the identical germline fixed light chain. The frequency of antigen-specific FlicAbs varied widely between animals which is not surprising given that on average 83% of the CDR3 clonotypes are unique to each animal (Figure 4). In addition, different immunization adjuvants result in significantly different proportions of ELISA-positive binders for certain antigens. Those antigens marked with an asterisk in Figure 4A show significant binding differences between Freund’s and Ribi adjuvants as measured by a Student’s *t*-test (*p*-value < 0.05).

We also compared antigen-specific binding behavior and sequence abundance of each FlicAb’s corresponding CDR3 clonotype. In Figure 4B, expressed CDR3 clonotypes are ranked by abundance, as measured by number of sequences within a clonotype, with ELISA binding status noted. Because we sampled proportionately across CDR3 clonotypes, 15% of the total 2,560 FlicAbs were selected from the most abundant CDR3 clonotype from a given animal, and a decreasing proportion of sequences were chosen from clonotypes of lower abundance. As shown in Figure 4C, 57% of FlicAbs from the most abundant CDR3 clonotype were antigen specific. The median percent of ELISA-positive FlicAbs in the top 10 most abundant CDR3 clonotypes is 43%, and the median percent positive in CDR3 clonotypes 11–20 is 31%. A Mann–Whitney *U* test for significance shows that these two medians are significantly different (*p*-value < 0.05).

### SHM in Germinal Center B-Cells

We performed NGS repertoire analysis on the heavy and light chain variable regions from the OmniFlic B-cell samples. By aligning each protein sequence from the heavy and light chain repertoires to the germline reference sequence, we calculated the distribution of SHM observed in the population of germinal center B-cells. Due to the multiple copies of the fixed light chain, we were able to confirm that 96% of the top 10 most prevalent light chain protein sequences were fully germline.

**Figure 5:**
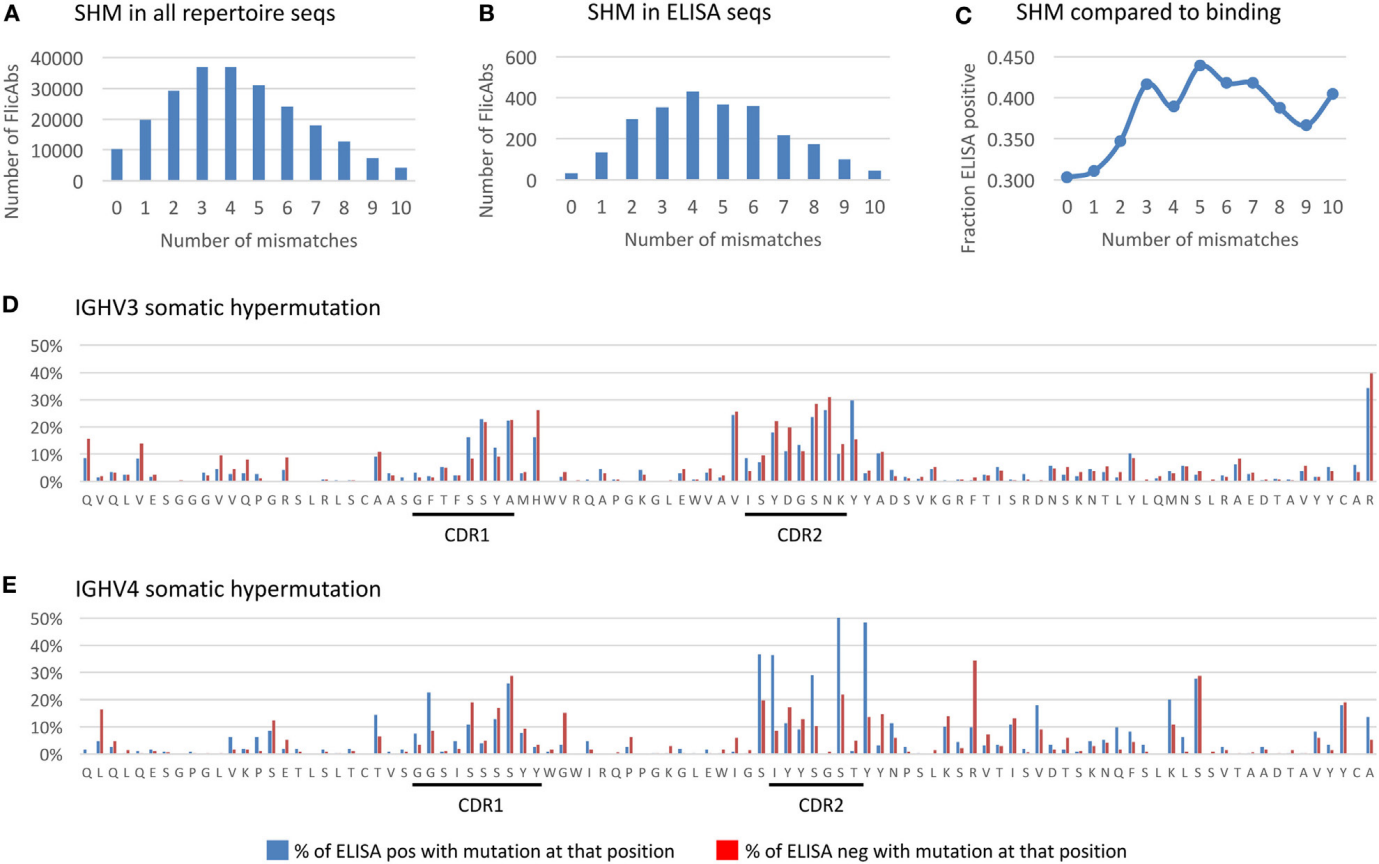
Somatic hypermutation in the heavy chain variable region. **(A)** The distribution of the total number of amino acid (AA) mutations in the heavy chain variable region is shown for the NGS repertoire data. The *x*-axis indicates the total number of AA mismatches compared with germline. The *y*-axis indicates the total number of FlicAbs. **(B)** The distribution of the total number of AA mutations in the heavy chain variable region is shown for the 2,560 sequences measured for antigen binding by ELISA. The labels are the same for panel **(A)**. **(C)** The frequency of ELISA-positive FlicAbs categorized by number of AA mismatches compared with germline is shown. The *y*-axis indicates the frequency of ELISA positive, and the *x*-axis category designates the number of mismatches compared with germline. **(D)** The AA mutation rates at each variable region position are shown for 1,770 sequences from the IGHV3 gene family. Blue bars indicate the percentage of ELISA-positive sequences with an AA mutation at that position. Red bars indicate the percentage of ELISA-positive sequences with an AA mutation at that position. The *x*-axis indicates the residue at each position of a representative germline sequence from the IGHV3 family. The *y*-axis indicates the percent of sequences with an AA mutation at a given position. **(E)** The AA mutation rates at each variable region position are shown for 615 sequences from the IGHV4 gene family. Labels are the same as panel **(D)**.

By contrast, across all of the samples tested, there were an average of 3.5 amino acid (AA) mutations from germline within the heavy chain variable region (Figure 5A). This calculation does not include the CDR3 junction due to insertions and deletions that make alignment to germline D-segments ambiguous. The distribution of SHM within the 2,560 monoclonal FlicAbs (Figure 5B) is similar to the distribution in the full repertoire. The equivalent distribution of SHM supports that the sample of FlicAbs we tested for antigen binding is representative of the repertoire as a whole.

We analyzed the FlicAb ELISA data in the context of SHM in the heavy chain variable region to see if the level of AA mutation was correlated with antigen-specific binding. As shown in Figure 5C, sequences with no AA mutation (100% germline) in the variable region showed the lowest percentage of antigen-specific FlicAbs (30%). By contrast, the highest percentage of antigen-specific FlicAbs (44%) comes from sequences with five AA mutations in the variable region. Interestingly, the percentage of antigen-specific binding increases with SHM up until a total of three mutations. From 3 to 10 AA mutations, the percentage of antigen-specific binding remains consistently around 40%. A Mann–Whitney *U* test for significance shows that the percentage of ELISA-positive FlicAbs with 0, 1, or 2 mutations is significantly lower than the percentage of positive FlicAbs with 3–10 mutations (*p*-value < 0.05).

For a high-resolution assessment of SHM patterns related to antigen binding, we measured the mutation frequency at each variable region amino acid position for 1,770 IGHV3 FlicAb sequences (Figure 5D) and 615 IGHV4 FlicAb sequences (Figure 5E) assayed by ELISA. As expected, the highest density of SHM occurs in the CDRH1 and CDRH2 regions with the CDRH2 region showing higher rates of mutation than CDR1. Interestingly, the N-terminal ends of FR2 and FR3 at the boundaries of CDRH1 and CDRH2, respectively, have AA mutation rates similar to the CDRs. This suggests that the traditional definitions of CDRH1 and CDRH2 boundaries may not encompass the full diversity and extent of the variable loops that define the paratope of the antibody. Further investigation is needed to determine if this is unique to OmniFlic.

We also stratified the SHM analysis by measuring the per AA residue mutation rates in ELISA-positive and -negative sequences. For the IGHV3 sequences, mutated AA residues in FR1 were approximately twice as prevalent in ELISA negative FlicAb sequences compared with ELISA-positive FlicAb sequences, while AA mutation frequencies in CDRH1 and CDRH2 were equivalent in the binding and non-binding sequences. By contrast, for the IGHV4 protein sequences, the mutated residues in CDRH2 were approximately twice as prevalent in ELISA-positive FlicAb sequences compared with ELISA negative FlicAb sequences. This suggests that AA mutations in CDRH2 in the IGHV4 gene family are preferentially selected for in antigen-specific FlicAb sequences.

## Discussion

Therapeutic antibody development is greatly facilitated by having a diverse source of antigen-specific antibodies from which to choose candidates for development and discovery methods that allow efficient evaluation of antibody diversity. In this study we show that the OmniFlic rat model produces a large sequence diversity of antibodies through the use of various VH genes and V–D–J recombination leading to a large number of distinct clonotypes despite each antibody using a common light chain. Furthermore, using next-generation sequencing to define the heavy chain repertoire enabled us to choose a diverse set of non-redundant sequences to express and functionally identify a large set of antigen-specific fixed light chain antibody proteins.

The naïve B-cell repertoire diversity of an organism is created by the random recombination of V, D, and J genes at the heavy chain locus and the recombination of V and J genes at the light chain locus. Naïve B-cells are exposed to antigen in germinal centers of lymphoid organs, and antigen-specific B-cells are positively selected for in the germinal centers (15). OmniFlic is the only known rodent model to use the IgKV3-15-JK1 fixed light chain, and it was unknown whether this single light chain would restrict VH-gene usage or reduce the diversity of clonotypes produced in the animal. Based on the frequency of VH-gene usage and clonotype diversity measured by NGS, we show in this study that the OmniFlic animal generates heavy chain diversity during a response to immunogen comparable to animals with unrestricted light chain use (9, 11, 23). Consistent with the large heavy chain diversity generated, there is very little overlap in the clonotypes produced between animals. Thus, including a large number of animals and multiple adjuvant cohorts in an immunization campaign is an effective way to increase the diversity of antibodies obtained.

In addition to measuring the sequence diversity generated in OmniFlic, we also characterized the immune response by measuring the antigen-specific serum titer in immunized animals and by measuring antigen-specific binding of isolated FlicAbs. Overall, 1,008 of the 2,560 FlicAbs tested (39%) were antigen-specific based on ELISA binding. There is a significantly higher proportion of antigen-specific FlicAbs found in the top 10 most abundant clonotypes compared with clonotypes ranked 11–20 in abundance. However, an outstanding question is why 43% of the most abundant clonotypes from each animal were ELISA negative presuming that abundance of a clonotype is indicative of positive selection to antigen. One potential explanation is that exposure to an immunogen is not the only factor driving B-cell selection and expansion within the germinal centers. The OmniFlic animals were not housed in a sterile environment, therefore we cannot exclude the possibility that a portion the animal’s immune response may be driven by exposure to environment antigens and pathogens. An alternative but not mutually exclusive explanation is that by capturing B-cell sequences at one point in time during the evolution of the B-cell repertoire in germinal centers, the immune system may not have selected against recent deleterious mutations. Likewise, antigen-specific sequences from low abundance clonotypes may represent newly arisen lineages that would have expanded further given more time. Therefore, while there is a higher frequency of antigen-specific FlicAbs in the most expanded CDR3 clonotypes, sequence abundance alone does not have a strong positive predictive value for antigen-specificity due to environment exposure and timing of B-cell harvest from lymph nodes. Interestingly, there was also no correlation between serum titer and percentage of antigen-specific FlicAbs (Figure S3 in Supplementary Material). This is likely due to the time lag between antigen-specific B-cell development in lymph node germinal centers and the accumulation of secreted antibody proteins in serum produced from mature plasma cells (24).

We also measured SHM in the context of antigen-specific binding. Lymph node germinal centers are sites of active B-cell affinity maturation through SHM and positive selection. The majority of mutations are detrimental and are selected against within the B-cell repertoire. However, the mutations that result in high affinity binding of the B-cell receptor are positively selected for by stimulating expression and expansion of individual B-cell clones. The OmniFlic animal model is unique in that it expresses the full human heavy chain locus and eight copies of a fixed rearranged light chain. We presume that allelic silencing does not occur on seven copies of the fixed light chain in a B-cell. Therefore, SHM of one fixed light chain gene will be diluted out by the expression from the other loci lacking that mutation thus negating the contribution of mutations in the fixed light chain to affinity maturation.

Due to the multiple copies of the fixed light chain, the process of affinity maturation in OmniFlic is dominated by SHM of the heavy chain variable region. Therefore, the antigen-binding characteristics of FlicAbs can be attributed to SHM patterns found in the heavy chain variable region. Overall, SHM resulted in amino acid substitutions enriched within and in close proximity to CDR1 and CDR2 of the VH gene as is observed with standard antibodies (25). We observed an interesting trend that the frequency of antigen-specific binding increased with up to 3 mutations in the VH region but did not increase from 3 to 10 average mutations. This suggests that more mutation in general does not necessarily lead to a higher likelihood of antigen-specific binding, and it is important to note that our observed relationship between antigen binding and mutation load could be related to the population of germinal center B-cells that contain a mixture of beneficial and deleterious mutations. Furthermore, the duration and degree of antigen exposure may also determine mutation load, and mature plasma cells or memory B-cells located in different parts of the body may have a different SHM profile (26, 27). As an example, human mAbs isolated from circulating memory B-cells from chronically infected HIV-positive patients have been reported to have 25–30 heavy chain mutations on average (28). Therefore, the developmental stage of the B-cells and the time course and overall dose of the antigen exposure can have a profound impact on SHM load (29). Our results reflect the SHM patterns seen in germinal center-derived B-cells following antigen exposure for 4 weeks.

In this study we have conducted a comprehensive analysis of the heavy chain sequence repertoire in a large number of immunized fixed light chain rodents. Cluster analysis of CDR3 clonotypes with 80% identity revealed an estimated 200 unique clonotypes per animal, of which two-thirds are unique to one lymph node. Animals immunized with the same antigens possessed an estimated 17% overlap of CDR3 clonotypes, indicating convergence, in contrast to those immunized with different antigens having 2% overlap. We determined that the V-gene usage in Flic animals matches that of humans and estimated that 57% of the most abundant CDR3 clonotypes were antigen specific. Finally, we determined the SHM frequencies of antigen-specific FlicAbs. Taken together, this study shows that the OmniFlic transgenic model is capable of producing a diverse collection of antigen-specific fully human fixed light chain antibodies that are excellent candidates for human therapeutics.

## Materials and Methods

### Creation of the OmniFlic Transgenic Rat

Our aim was to derive a transgenic rat strain expressing a diverse human heavy chain repertoire combined with a single fixed light kappa chain with minimal hypermutation. OmniFlic was created using the same HSD:Sprague Dawley genetic background containing the rat IgH, Igκ, and Igλ triple knockout described by Osborn et al. (9). The human IgH V genes (IGHV4-39 through IGHV6-1), human J genes, human D genes and rat constant region genes were introduced using the same BACs (BAC3, BAC6, and Annabel) described by Osborn et al. (9).

We constructed a rearranged human Igk L-chain (RK) by adding IgKV3-15 to IgKJ1. We chose IgKV3-15 and IgKJ1 because this rearrangement was observed frequently in natural human repertoires (8%) and paired with a wide variety of heavy chain variable genes (11). A self-replicating shuttle vector, termed pCAU, efficiently working in both *Saccharomyces cerevisiae* and *E. coli*, was constructed based on pBelo-CEN-URA published previously (30). In brief, *ARS109* was amplified from *S. cerevisiae* genomic DNA, with an *ApaL*I site followed by *AsiS*I and a *SexA*I introduced into either end. The fragment was digested with *ApaL*I and *SexA*I and ligated with pBelo-CEN-URA digested with the same restriction enzymes to yield pCAU. This vector contains *S. cerevisiae CEN4, URA3*, and *ARS109* in the pBeloBAC11 backbone (New England BioLabs).

A 4.2 kb fragment spanning from 4 kb upstream of human IGKV3-15 to the 3′ end of IGKV3-15 was amplified using diluted BAC RP11-156D9 (Life Technologies) as template. A 1.3 kb fragment comprising a 25 bp sequence at the 3′ end of IGKV3-15 followed immediately by human IGKJ1 to the intergenic region between IGKJ4 and J5 was amplified, and diluted BAC RP11-344F17 (Life Technologies) as template. Equal amounts of the above two fragments were mixed and a fusion PCR was performed to produce a joined 5.5 kb fragment. This joined fragment contained the rearranged IGKV3-15-JK1 (RK) exon. Subsequently, the circular YAC (cYAC) containing RK was assembled in *S. cerevisiae* with the following three overlapping fragments: the joined fragment above, a 40 kb *Afe*I to *Pac*I fragment encompassing the 3′ region of Human J_K_s, C, and KDE which was cut out and purified from RP11-344F17, and the pCAU vector. Finally, the resulting cYAC was converted into a BAC, termed RK, as described previously. Extensive restriction mapping and sequencing were performed to confirm the sequence authenticity of the resulting BAC. A 46 kb AsiSI fragment containing the RK (IGKV3-15-JK1) can be cut out and purified.

Integration of RK by DNA microinjection into fertilized oocytes produced 21 founders with between 1 and 20 transgene copies (data not shown). Breeding of a selected RK founder animal with a human heavy chain founder animal resulted in OmniFlic progeny with different RK copy numbers. Breeding for all transgenic and KO features showed stable transmission.

Initial validation experiments in OmniFlic showed that there was normal class switching to IgG and normal Ig expression levels in the serum using conventional adjuvants and immunization protocols (31) (data not shown). Average antigen-specific serum titers of 1:10,000 to 1:40,000 were also similar to transgenic rats carrying the same IgH locus. We found the highest frequency of RK devoid of SHM in an OmniFlic line with eight copies of the RK transgene, and we used this line for all subsequent studies.

### Summary of Study Design and Data Analysis

After validation of the OmniFlic model and selection of the line showing the fewest expressed light chain mutations, we undertook a series of immunizations and monoclonal FlicAb discovery campaigns. We immunized 75 OmniFlic animals, each with 1 of 9 different protein antigens. From each of these animals we harvested two draining lymph nodes, one from each side of the animal. We analyzed each lymph node separately for a total of 150 independent samples.

We dissociated primary lymphocytes from each lymph node and isolated RNA from these cells for NGS analysis of the expressed IgG heavy chain repertoire. Thus, downstream analysis of the heavy chain repertoire data is focused largely on germinal center-derived B-cells along with other IgG lymphocytes present in the lymph nodes. Our analysis of the repertoire data focused on quantifying IGHV-gene usage, measuring VDJ clonotype diversity between lymph nodes and between animals, and measuring patterns of SHM. Furthermore, we expressed a total of 2,560 monoclonal FlicAbs derived from the repertoire sequences, measured protein binding by ELISA, and analyzed functional binding data in the context of clonotype size and SHM. Overall study design and analysis work flow is summarized in Figure 1.

### Immunization, Variable Region Amplification, and Next-Generation Sequencing

We immunized OmniFlic animals according to a standard 48-day protocol. For each antigen, we immunized three to four animals with Complete Freund’s adjuvant (one prime injection and three boosts) and three to four animals with TiterMax/ribi adjuvant (one prime and six boosts) with bilateral injections in the hind legs of the animal. We collected a pre-harvest bleed at day 35 of the immunization protocol from each animal to assess antigen-specific serum titer. The nine different antigens were all human recombinant extracellular domains of cell surface proteins.

At day 48 of the immunization protocol, we harvested lymphocytes from the left and right draining lymph nodes of immunized Flic animals and processed each lymph node separately. We washed dissociated lymphocytes with PBS and pelleted the cells by centrifugation at 1,000 × *g* for 10 min. We removed the supernatant and froze the remaining cell pellet. We then isolated total RNA from each cell pellet using the RNeasy kit according the manufacturer’s protocol (Qiagen catalog number: 74034). We then performed first strand cDNA synthesis and 5′ RACE by PCR amplification of the full Ig heavy chain or Ig kappa light chain variable regions according to previously published protocols (32, 33).

We isolated the resulting product of approximately 500 bp and purified using the QIAquick gel extraction kit according to the manufacturer’s protocol (Qiagen catalog number: 28704). To multiplex multiple samples on a single next-generation sequencing run we added sample index labels to each sample by primer extension using a previously described index PCR reaction (34). We then pooled the resulting indexed samples to create our sequencing library and we sequenced the library on the Illumina MiSeq platform with 2 × 300 paired-end reads.

### Analysis of NGS Sequencing Depth

We generated approximately 100,000 paired-end reads for each sample sequenced. To determine the total number of CDR3 clonotypes present in the sample based on the number of CDR3 clonotypes identified at this sequencing depth, we conducted four technical replicate sequencing runs from one lymph node sample. These experiments resulted in an average of 112 unique CDR3 clonotypes per experiment. We then measured the overlap of CDR3 sequences between each pairwise technical replicate. The average overlap between pairwise comparisons was 96. Mathematically, these results can be modeled as a twice-replicated counting experiment in which some number of entities (112 in this case) is chosen from a larger population. From the number of entities chosen repeatedly in the two separate counting experiments, the actual size of the total population can be reasonably inferred.

$\left(\begin{array}{c} n\\ k_{1} \end{array}\right)$ and $\left(\begin{array}{c} n\\ k_{2} \end{array}\right)$ and want to infer the most likely value of *n* from the value of *y* = *k*_1_ ∩ *k*_2_, where *k*_1_ and *k*_2_ are uniquely identifiable objects (with *k*_1_ = *k*_2_).

To achieve this goal, we conducted a computer simulation using Python and the NumPy (numerical python) library in which we varied the values of *n* and *k*_1_, *k*_2_. For each distinct set of *n* and *k*_1_, *k*_2_ values, we repeated the simulation 5,000 times and averaged the resulting overlap. As the population size *n* varies, the average overlap value *y* = *k*_1_ ∩ *k*_2_ changes, and we can view the average overlap y as a function of the population size. Our simulations can be generalized by the following equation:

$$y(n)\text{=}k^{2}n^{-1},$$

where *y* = average number of objects found in both repeated samplings; *k* = the number of objects sampled in each individual experiment; and *n* = the total size (number of distinct objects) in the population being sampled.

Based on this equation, we determine the appropriate value of *n* from

y=96(CDR3 clonotypes found in both technical replicates),

k=112(total CDR3 clonotypes sampled in each experiment),

and find a corresponding value of *n* = 131.

Thus, our technical replicate results suggest a likely starting population of 131 unique CDR3 clonotypes. We therefore calculate our sampling efficiency as follows:

112 sampled/131 total available=85.5%.

### NGS Clonotype Analysis

For the NGS-based clonotype analysis, we downloaded and processed all paired fastq reads for each sample. Each sample was covered by approximately 100,000 paired reads on average. A first pass quality control of the sequence was performed to eliminate artifactual sequences with homopolymer runs of 30 or more bases. We also performed a permissive alignment to all human V-gene framework 1 sequences to keep only those sequences with at least 20 aligned nucleotides derived from the human Ig locus. After we applied the sequence QC filters described, we first merged the forward and reverse paired reads by aligning the paired reads using the FLASH package,^1^ and we kept all reads that were successfully merged. After merging the fastq reads, we then determined the longest open reading frame for each merged read and generated an output of the translated amino acid protein sequence encoded by the open reading frame. We then aligned all of the protein sequences to the set of human germline IGHV genes from IMGT using IGBLAST.^2^ Based on the protein alignments and the IMGT coordinate system, we then determined the framework and CDR regions of the full heavy chain variable region protein sequence. Based on the CDR and framework annotation, we then determined the CDR3 sequence contained in each protein sequence derived from the paired sequence reads. We then used agglomerative clustering to cluster the full set of CDR3 protein sequences for each sample at an 80% similarity threshold and recorded the total number of reads in each cluster. We define a clonotype as the cluster of CDR3 protein sequences clustered at 80% similarity. We calculated the total number of CDR3 clonotypes as all of the CDR3 clonotypes comprised of five or more paired sequence reads. A summary of the NGS sequence metrics derived from the samples analyzed can be found in Table S1 in Supplementary Material.

To compare the overlap of CDR3 clonotypes between lymph node samples or samples from different animals, we performed an all-by-all comparison of the consensus sequence from each CDR3 clonotypes and used the Wagner–Fischer algorithm to calculate the Levenshtein distance between two CDR3 consensus sequences.^3^ Two sequences were said to match when the Levenshtein distance between the two sequences were less than 20% the length of the longest sequence. This criterion for matching is consistent with the two sequences belonging to the same CDR3 clonotypes based on 80% similarity.

We calculated the polarization, or skewed, representation of CDR3 clonotypes by calculating the percentage of total number reads in a sample that are contained in each CDR3 clonotype. We then ranked the CDR3 clonotypes based sequence read abundance for each sample. We then calculated the mean and 25th and 75th quartile values of corresponding ranked CDR3 clonotypes across all samples using the BoxPlotR package available at: http://shiny.chemgrid.org/boxplotr/.

### Ig Heavy Chain Variable Gene Usage

To calculate the Ig heavy chain variable gene usage, we used the alignment of amino acid sequence to the germline reference sequences from IMGT described earlier. We assigned the IGHV gene to each amino acid sequence based on the most similar match to the reference set of germline sequences at IMGT. For each IGHV gene, we calculated the percentage of amino acid sequences from a given sample assigned to that IGHV-gene based on the total number amino acid sequences derived from that sample. The IGHV-gene usage was then assembled into a table where each row was an individual sample and each column was an IGHV gene.

### SHM Analysis

To calculate the SHM in the Ig heavy chain variable region, we used the alignment of the IGHV amino acid sequence to the closest germline reference sequence from IMGT that was described earlier. For each variable region amino acid sequence, we calculated the total number of mismatches compared with germline. We also calculated the total number of mismatches in the 2,560 sequences measured by ELISA. We binned the ELISA sequences by those that had 0, 1, 2, 3, 4, 5, 6, 7, 8, 9, or 10 or more mismatches compared with germline. Then we calculated what fraction of the sequences in each bin were ELISA positive.

Furthermore, for 1,770 IGHV3 and 615 IGHV4 variable gene sequences that were assayed by ELISA, we also calculated the mismatches per amino acid position in the variable regions of those gene families. A multiple sequence alignment was constructed for the 1,770 IGHV3 sequences plus the corresponding germline reference sequences. Separately, a multiple sequence alignment was constructed for the 615 IGHV4 sequences plus the corresponding germline reference sequences. For each amino acid position in each sequence, we recorded whether the amino acid residue was germline or mutated and whether that sequence was ELISA positive or negative. We collected these results in aggregate for IGHV3 and IGHV4 separately and calculated the percent of ELISA-positive sequences with a mutation at each position, and calculated the percent of ELISA negative sequences with a mutation at each position.

### Recombinant Antibody Expression and ELISA Assays

We chose a representative sampling of heavy chain variable sequences from the diversity of clonotypes identified in each animal to express and measure antigen binding by ELISA. We individually cloned a total of 2,560 heavy chain variable regions into a heavy chain expression vector containing a leader peptide sequence and the human IgG1 Fc region. Detail methods on variable region cloning and the vectors used were described previously (33). We sequence validated the 2,560 individual heavy chain clones using Sanger sequencing on an ABI 3730xl DNA Analyzer platform to ensure they were the correct sequence and were cloned in frame with the leader peptide and Fc region. We also created and sequence validated a kappa light chain expression vector that contains the light chain variable region that corresponds to the germline sequence of the rearranged kappa light chain in the OmniFlic animal. We individually transformed each expression vector into TOP10 chemically competent *E. coli* according to manufacturer’s protocols (Thermo Fisher catalog number C404003), grew them for 24 h in 2 mL of LB culture media and purified them in 96-well format using the Qiagen Plasmid Plus 96 Kit according to the manufacturer’s protocol (Qiagen catalog number: 16181). We assessed the quantity and purity of the purified expression vectors by calculating the 260 and 280 nM absorbance ratio. After purification and spectroscopic analysis, we then normalized the concentration of each vector.

We recombinantly expressed the monoclonal FlicAbs by first mixing equal amounts of each heavy chain expression vector with the common light chain expression vector using previously described methods (33). We transfected each of the individual heavy and light chain vector mix in 293 cells in 96-well format using previously described methods (33). After transfection and expression, we then harvested and clarified the cell culture supernatants by centrifugation at 2,000 × *g* for 10 min. We measured the concentration of the FlicAb protein contained in the clarified supernatant using protein A biosensor tips on the Octet using a standard curve of known antibody concentration (ForteBio catalog number 18-5013). After confirming antibody concentration of at least 0.2 μg/mL in the cell culture supernatant, we then measured protein binding by ELISA using the antibody-containing supernatant.

We performed high-throughput ELISA assays to measure antigen-specific binding of the monoclonal FlicAbs. First, we coated 96-well polystyrene microtiter plates with 5 µg/mL of the recombinant protein of interest in BupH Carbonate-Bicarbonate buffer. The recombinant protein used in the ELISA was the same protein used to immunize the OmniFlic animals. We then incubated 8–12 h overnight at 4°C overnight. We then blocked antigen-coated wells with 1% milk/TBST for 1 h at room temperature. After blocking, we diluted FlicAb-containing supernatants 1:100 in 1% milk/TBST, added the supernatants to blocked antigen-coated wells, and incubated for 1 h. After the 1 h incubation with primary antibody, we added a secondary horseradish peroxidase-conjugated antihuman IgG antibody and allow to incubate for 0.5 h. We then added ELISA chemiluminescent substrate to each well and measured the chemiluminescent signal on a plate luminometer. We expressed the binding signal as the ratio of experimental well signal over background signal, where background signal is determined by the average signal from negative control cell culture supernatants. The negative control cell culture supernatants were harvested from wells of untransfected 293 cells cultured in parallel with transfected cells.

## Ethics Statement

This study was carried out in a research facility registered with the California Department of Public Health and, as such, the animal care and use program is required to adhere to the NIH Guide for the Care and Use of Laboratory Animals. We certify that all work was performed using Institutional Animal Care and Use Committee (IACUC) protocols that have been reviewed and approved by our IACUC committee.

## Author Contributions

KH conducted NGS and managed molecular biology; SA contributed to overall study design and manuscript preparation; LD, HO, and KD contributed to molecular biology and binding assays; AB contributed to NGS analysis; MB contributed to creation of transgenic animal; MO and BM contributed to transgenic animal; BB, SI, WS, and OV contributed to analysis; SC, DP, and UR contributed to binding assays; KD contributed to molecular biology and binding assays; BJ, US, PP, and HU contributed to expression; RB contributed to creation of transgenic animal and overall study design; NT contributed to overall study design and analysis and wrote the manuscript.

## Conflict of Interest Statement

All authors are employees of Teneobio with equity interests.

## Footnotes

^1^https://ccb.jhu.edu/software/FLASH/ (Accessed: Feb 2017).

^2^https://www.ncbi.nlm.nih.gov/igblast/ (Accessed: Feb 2017).

^3^http://search.cpan.org/~davidebe/Text-WagnerFischer-0.04/WagnerFischer.pm (Accessed: Feb 2017).

## Supplementary Material

Figure S1 Overlap of CDR3 clonotypes between animals. An all-by-all comparison matrix of the percent of CDR3 clonotypes that are found in common between two animals is shown as a heat map. Blue indicates 0% overlap, and red indicates 100% overlap.

Figure S2 Clonotype polarization. The degree and variation of polarization across 75 animals is shown as box plots for CDR3 clonotypes ranked by abundance. The individual sequences in the most abundant CDR3 (rank1) comprise between 10 and 90% of the total sequences generated in a single animal. The *y*-axis shows the fraction of total sequences. The *x*-axis category designates the abundance rank of the CDR3 clonotype. The inset scatterplot shows the relationship between total number of unique CDR3 clonotypes in an animal and the fraction of sequences contained in the most abundant CDR3 clonotype. The *x*-axis of the scatterplot indicates the total unique CDR3 clonotypes in each animal. The *y*-axis indicates the fraction of all sequences contained in the most abundant CDR3 clonotype in each animal.

Figure S3 ELISA binding compared with antigen-specific serum titer. The percent ELISA-positive FlicAbs in a single animal is compared with the antigen-specific serum titer in the same animal in this scatterplot. The *x*-axis indicates the percentage of ELISA FlicAbs that are ELISA positive in an animal compared with the total FlicAbs tested in the animal. The *y*-axis indicates the antigen-specific serum titer.

Table S1 NGS sequence metrics. Basic sequence metrics for the samples analyzed are shown in this table. Column 1 indicates the unique sample identifier. Column 2 shows the total number of raw read pairs (forward and reverse reads) collected for each sample. Column 3 shows the total number of read pairs that pass the quality control filters described in the methods. Column 4 shows the percent of the QC forward and reverse read pairs that were successfully merged. Column 5 shows the total number of productive VH open reading frames identified in the merged reads that were used in downstream analyses.
