# Supplementary figures and images for: Sequence-Based Discovery Demonstrates That Fixed Light Chain Human Transgenic Rats Produce a Diverse Repertoire of Antigen-Specific Antibodies
Source: Front Immunol. 2018 Apr 24;9:889. doi: 10.3389/fimmu.2018.00889 (PMC5928204; doi:10.3389/fimmu.2018.00889)

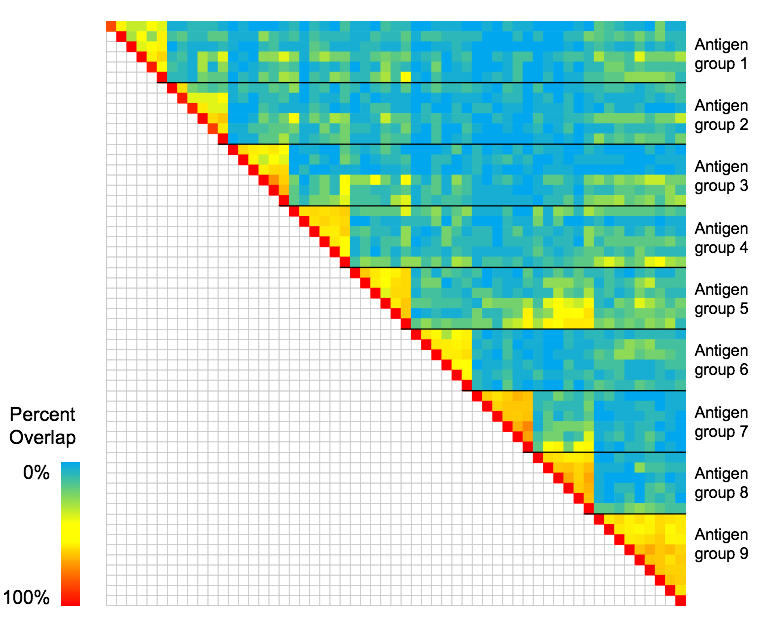

Supplement: Figure S1 — Overlap of CDR3 clonotypes between animals. An all-by-all comparison matrix of the percent of CDR3 clonotypes that are found in common between two animals is shown as a heat map. Blue indicates 0% overlap, and red indicates 100% overlap. [file Image_1.jpeg]

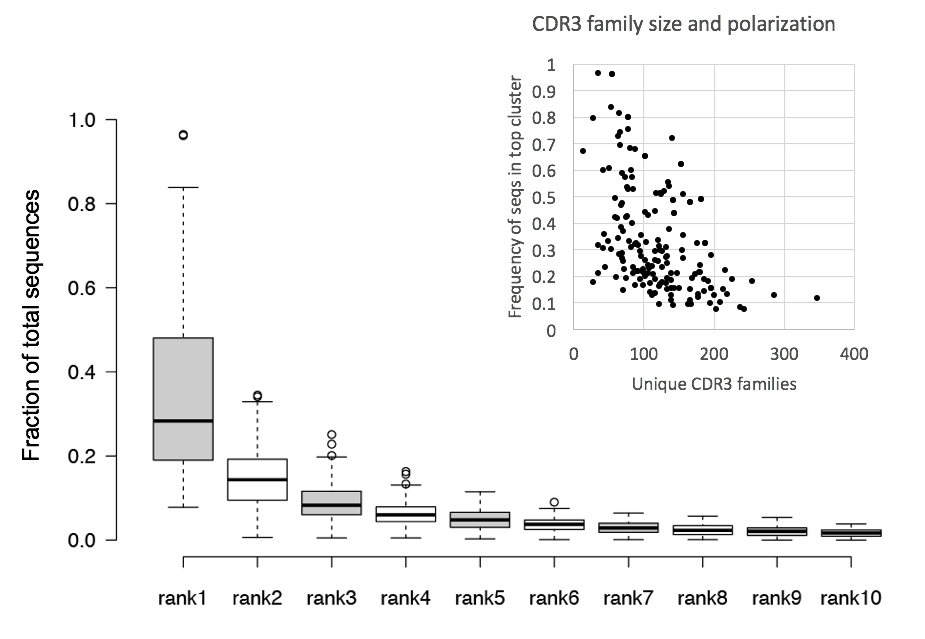

Supplement: Figure S2 — Clonotype polarization. The degree and variation of polarization across 75 animals is shown as box plots for CDR3 clonotypes ranked by abundance. The individual sequences in the most abundant CDR3 (rank1) comprise between 10 and 90% of the total sequences generated in a single animal. The y-axis shows the fraction of total sequences. The x-axis category designates the abundance rank of the CDR3 clonotype. The inset scatterplot shows the relationship between total number of unique CDR3 clonotypes in an animal and the fraction of sequences contained in the most abundant CDR3 clonotype. The x-axis of the scatterplot indicates the total unique CDR3 clonotypes in each animal. The y-axis indicates the fraction of all sequences contained in the most abundant CDR3 clonotype in each animal. [file Image_2.jpeg]

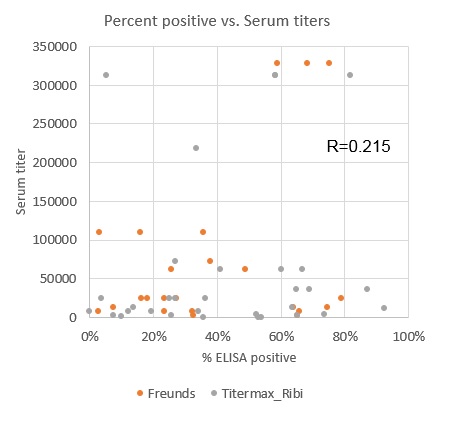

Supplement: Figure S3 — ELISA binding compared with antigen-specific serum titer. The percent ELISA-positive FlicAbs in a single animal is compared with the antigen-specific serum titer in the same animal in this scatterplot. The x-axis indicates the percentage of ELISA FlicAbs that are ELISA positive in an animal compared with the total FlicAbs tested in the animal. The y-axis indicates the antigen-specific serum titer. [file Image_3.jpeg]
